# The therapeutic potential of mesenchymal stem cells in treating osteoporosis

**DOI:** 10.1186/s40659-021-00366-y

**Published:** 2021-12-20

**Authors:** Tianning Chen, Tieyi Yang, Weiwei Zhang, Jin Shao

**Affiliations:** 1 Ningxia Medical University, Yinchuan, 750004 Ningxia Hui-Autonomous Region China; 2 Department of Orthopedics, Pudong New Area Gongli Hospital, School of Clinical Medicine, Shanghai University, Shanghai, 200135 China; 3 Department of Urology, Renji Hospital, School of Medicine, Shanghai Jiao Tong University, Shanghai, 200127 China

**Keywords:** Osteoporosis, Mesenchymal stem cells, Therapeutic potential

## Abstract

Osteoporosis (OP), a common systemic metabolic bone disease, is characterized by low bone mass, increasing bone fragility and a high risk of fracture. At present, the clinical treatment of OP mainly involves anti-bone resorption drugs and anabolic agents for bone, but their long-term use can cause serious side effects. The development of stem cell therapy and regenerative medicine has provided a new approach to the clinical treatment of various diseases, even with a hope for cure. Recently, the therapeutic advantages of the therapy have been shown for a variety of orthopedic diseases. However, these stem cell-based researches are currently limited to animal models; the uncertainty regarding the post-transplantation fate of stem cells and their safety in recipients has largely restricted the development of human clinical trials. Nevertheless, the feasibility of mesenchymal stem cells to treat osteoporotic mice has drawn a growing amount of intriguing attention from clinicians to its potential of applying the stem cell-based therapy as a new therapeutic approach to OP in the future clinic. In the current review, therefore, we explored the potential use of mesenchymal stem cells in human OP treatment.

## Introduction

The concept of osteoporosis (OP) can be traced back to 1885 when Gustav Pommer, German pathologist, defined the distinction between OP and osteomalacia [1]. Subsequently, of OP the predisposing factors, general aetiology and pathogenesis, clinical presentation, prevention, treatment, and prognosis were deeply studied in the twentieth century [2, 3]. In the 1990s, a clear definition of OP was put forward to be recognized worldwide [4, 5]. Currently, it is believed that OP is a multifactorial, systemic metabolic bone disease characterized by the deterioration of bone mass and microstructure destruction, which lead to decreased bone mineral density (BMD), increased bone fragility and the risk of bone fracture [6]. The occurrence and progression of OP are believed to be closely related to aging, with its incidence being the highest in elderly men and postmenopausal women. As previously reported, an estimated 54 million American women and men aged over 50 suffered from OP and osteopenia [7], and in China the OP-stricken population would reach over 120 million by 2050 [8].

In the treatment of OP, the primary goal is to maintain the balance between bone metabolism and reduction of bone loss. At present, the clinically administered medications to treat OP are divided into three categories according to pathogenesis: basic supplements, antiresorptive agents and bone formation-accelerating agents [9]. Vitamin D, as a basic supplement and was demonstrated not to have a therapeutic effect on fracture or BMD [10]. The antiresorptive agents to treat OP include bisphosphonate, which has a variety of side effects [11]. In recent years, the stem cell therapy for OP has been reported to reduce bone loss and decrease the patient’s vulnerability to fractures [12]. Stem cells, derived from embryos, fetuses or adults with unlimited self-renewal, proliferation and differentiation ability under specific conditions, can be classified into totipotent SCs (TSCs), pluripotent SCs (PSCs) and unipotent SCs (USCs) according to their differentiating potential [13]. As pluripotent stem cells, mesenchymal stem cells (MSCs) show relatively high versatility and differentiate into multipotent bone, cartilage and adipose tissue cells; these cells have become the most appropriate source for stem cell-based therapy [14].

The goal of our research was to have a comprehensive review of the recent advances in treating OP with mesenchymal stem cell therapy. In particular, our review focused on the preclinical and clinical researches on the use of bone marrow-derived mesenchymal stem cells (BM-MSCs), adipose tissue-derived MSCs (AD-MSCs), and umbilical cord-derived MSCs (UC-MSCs) for OP treatment.

## Physiopathology of osteoporosis

OP is a complex metabolic disease that is associated with risk factors such as high BMI, history of smoking and drinking, age at menopause, and postmenopausal status [15, 16]. High BMI caused by obesity was previously considered to be conducive to maintaining bone health and reducing the risk of fracture [17]. Recent studies have shown that obesity can cause a harmful effect on bone metabolism, and the weakened bone strength may be related to the location of fat accumulation. In obesity, although the overall fat mass increases significantly, the part of lean body weight gain has been proved to have a beneficial effect on BMD [18]. We believe that this phenomenon is related to the increase of bone mechanical load. However, high body fat rate and high waist circumference are considered to be associated with low BMD and osteoporotic fractures [19, 20]. Peptide hormones produced by adipose tissue are involved in bone metabolism [21]. Leptin plays a two-way regulatory role in bone metabolism. On the one hand, leptin can stimulate the differentiation of osteoblasts and inhibit the differentiation of pluripotent stem cells into adipocytes to promote osteogenesis; on the other hand, bone formation can be decreased by reducing insulin resistance and inhibiting bone anabolism [21, 22]. Additionally, the increase of fat content is related to the increase of androgen to estrogen, which has a positive effect on bone metabolism. Therefore, when discussing the contradictory relationship between obesity and OP, the protective and harmful effects of fat on bone must be considered simultaneously [23].

With pain being the predominant symptom, OP facilitates compression fractures of the vertebrae and traumatic fractures of the femoral neck in its victims, thus leading to a significant decline in the quality of life. In fact, the pathophysiological mechanism of OP is sophisticated and multifactorial. As a consequence of aging and/or oestrogen deficiency, the disorders of bone metabolism act as the principal pathophysiologic mechanism of primary OP. Under normal physiological conditions, the homeostasis of bone metabolism is mainly regulated by osteoblasts, osteoclasts, and osteocytes. Osteocytes are the most abundant cells in bone tissues, accounting for more than 90% there, and seeming to play an indispensable role in signal transduction in bone metabolism [24]. Osteoclasts, which are multinucleated cells that originate in haematopoietic myeloid cells in bone marrow (BM), can absorb mineralized bone matrix, as the primary cells involved in bone destruction in OP. Osteoblasts, which are responsible for the formation of new bone have three main processes involved; firstly, extracellular matrix proteins are synthesized by osteoblasts, and subsequently, extracellular matrix proteins are covered by a layer of calcium hydroxyapatite crystals within the next few months, thereby mineralizing the matrix, and ultimately, bone remodelling occurs.

Actually, numerous signalling pathways are involved in the regulation of bone metabolism; the key ones mainly refer to the receptor activator of nuclear factor-kappa B (RANK)-RANK ligand (RANKL) and Wnt/β-catenin signalling pathway. In the 1990s, it was discovered that the RANKL/RANK/Osteoprotegrin (OPG) signalling pathway is the key to the regulation of osteoclast generation, providing a theoretical basis for the research on and development of new anti-reabsorption drugs such as denosumab [25]. RANKL combines with RANK on the cell membrane surface to regulate the recruitment and differentiation of osteoclasts, thus playing a role in inducing the differentiation and maturation of osteoclasts and promoting bone absorption. OPG, a soluble trap receptor secreted by osteoblasts, plays a competitive role in binding RANKL along this pathway. OPG shows a stronger affinity with RANKL than does RANK, thereby inhibiting osteoclast differentiation, activation, and maturation by blocking binding between RANKL and RANK, which significantly reduces the ability of RANK to promote osteoclast formation. Furthermore, OPG can promote bone formation by activating osteoblasts, thus exerting an inhibitory effect on bone resorption [26].

As the major regulator of osteoblast-mediated bone formation, the canonical Wnt/β-catenin signalling pathway, once activated, promotes dishevelled protein expression and activation and mediates GSK-3β phosphorylation, facilitating the stable maintenance and continuous aggregation of β-catenin in target cells. β-catenin is known to enter the nucleus from the cytoplasm; when its concentration reaches a certain level in the cytoplasm, it combines with LEF/TCF family members to recruit bcl9 and other related factors to positively regulate the expression of target genes, promoting the proliferation and differentiation of bone marrow mesenchymal stem cells into osteoblasts. Simultaneously, activated Wnt signalling can promote the secretion of OPG in the RANK/RANKL/OPG signalling pathway, thus inhibiting the differentiation, activation, maturation of osteoclasts, and promoting bone formation [27].

## The current treatment of OP

The treatment involving nonpharmacological interventions is the primary approach to those who are at the early stage of OP. This approach is mainly taken in the form of ensuring adequate daily calcium, vitamin D, and protein intake; administering an appropriate amount of weight-bearing physical exercise to maintain or improve physical quality; and facilitating appropriate lifestyle changes such as smoking cessation and alcohol consumption moderation. As the disease progresses, pharmacological interventions become inevitable; calcium and vitamin D are routinely recommended for all patients as OP treatment. Both substances are essential for the stabilization of the normal bone internal environment so as to reduce the rate of bone loss and fracture risk.

Previous study has shown that additional intake of vitamin D and calcium supplements each day can significantly improve the calcium balance in postmenopausal women, resulting in a significant increase in BMD at the femoral neck exerting favourable effects on glucose and lipid level [28]. At present, a variety of medications are clinically prescribed to treat OP, which mainly refer to bisphosphonates, parathyroid hormone (PTH) and its analogues, a selective oestrogen-receptor modulator (SERM, raloxifene), a human monoclonal antibody against RANKL (denosumab), a humanized monoclonal antibody against sclerostin (romosozumab) and a cathepsin K inhibitor (odanacatib). As the most widely administered bone resorption inhibitor, bisphosphonates can induce osteoblasts to secrete inhibitors, exerting cytotoxic effects on osteoclasts, inhibiting the activation of osteoclasts, and restraining bone resorption. Moreover, bisphosphonates which combine with calcium phosphate are adsorbed on the surface of bone hydroxyapatite crystals to prevent the loss of calcium in bone, thus reducing the incidence of lumbar and hip fractures in patients with OP [29]. Bisphosphonates, widely used in the clinic, definitely have a curative effect, but with some side effects, as indicated in the previously reported evidence that they may cause typical osteonecrosis of femoral fracture and jaw [30, 31]. Teriparatide (recombinant human PTH [1–34]), the first bone anabolic agent approved by the FDA, can effectively promote bone formation on a short-term course [32], but increase the occurrence of osteosarcoma in the preclinical rat models on a long-term and high-dose course [33]; therefore, the treating course of teriparatide has been limited to 24 months by the FDA. Selective oestrogen-receptor modulators, similar to oestrogen replacement therapy (ERT), are mainly taken to prevent bone loss in postmenopausal women; however, the use of these drugs increases the risk of breast cancer, cardiovascular events, stroke, and pulmonary embolism [34]. Denosumab, a human monoclonal antibody against RANKL, is capable of competitively binding to the RANKL protein, as in the case of OPG, inhibiting the differentiation and activation of osteoclasts mediated by the RANKL/RANK/OPG signalling pathway, reducing osteoclastic activity and improving bone mass and bone density [35].

As indicated in a clinical study, OP patients receiving denosumab could experience a 68% reduction in vertebral fracture and a 40% decrease in hip fracture [36]. The most common complication of the denosumab treatment is hypocalcaemia, and another serious one, osteonecrosis of the jaw, which was the first to be reported in the patients on bisphosphate [37]. In the FREEDOM experiment, of 4550 patients who received denosumab 5 developed severe complications of osteonecrosis in the jaw [38]. Romosozumab, a humanized monoclonal antibody against sclerostin, can bind to osteosclerotin in vivo, inhibiting its binding with LRP5/6 and other proteins of the low-density lipoprotein-related receptor family, enhancing the Wnt signalling pathway, significantly increasing bone mass formation, and reducing bone resorption, which exerts a dual regulatory effect [39]. When compared with other monoclonal antibody medications including denosumab, romosozumab does not increase the risk of fracture after drug withdrawal and have a significant effect on the incidence of tumours in a rat model [40], but it may be more prone to causing local reactions at the injection site such as discomfort, pain, erythema, rash, haematoma or bleeding [41]. Cathepsin K, a lysosomal protease that is highly expressed in osteoclasts, plays a key role in the degradation of bone matrix proteins, and cathepsin K inhibitors show a similar efficiency as bisphosphonates in increasing BMD and decreasing the risk of fragility fractures [42]. Odanacatib inhibits bone resorption without reducing the numbers of osteoclasts or inhibiting bone formation, and is well tolerated without significant drug-related side effects [43].

In the past few decades, an extraordinary understanding has been achieved on the biology of bones, so have remarkable progresses been made in the development of OP medications. However, further researches are still needed on the development of new drugs and therapies with fewer side effects. Since stem cell therapy is considered a promising new therapeutic strategy for restoring normal tissue structure and delaying disease progression. OP therapy based on mesenchymal stem cells can be of great interest.

## The characteristics of MSCs

For the first time in 1968, Friedenstein et al. isolated MSCs from bone marrow [44]. As indicated in the subsequent studies, MSCs exhibit immune and nutritional activity and high in vitro self-renewal and multilineage differentiation capabilities [45], and they also express and secrete numerous bioactive factors, including cytokines [46], growth factors and chemokines, which are involved in the paracrine activity of MSCs [47]. Adult MSCs have been found to exist in almost all tissues, such as bone marrow, umbilical cord and placenta, adipose tissue, peripheral blood, and endometrial tissue. Not only can they differentiate into a variety of connective tissues, such as bone, muscle, adipose tissue, and cartilage, but they can also differentiate into nonmesodermal lineage cells, such as hepatocytes, neuron-like cells and pancreatic cells; moreover, they can differentiate into other types of cells under suitable conditions [48].

As indicated in the previously reported studies, many potential MSC surface markers are related to stem cell characteristics, including CD73, CD90, and CD105, but from this list are missing CD19, CD45, CD34, CD14, CD79α and HLA-DR. There are significant differences in the expression of these markers on MSCs from different sources [49]. Human bone marrow, umbilical cord blood, and adipose tissue have been the most used sources of adult MSCs [50]. Among these cell sources, BM-MSCs have been extensively studied in the context of tissue regeneration and repair due to their efficient differentiation ability. Adipose tissue is especially abundant and readily available in our body, which makes it the safest and most reliable site for stem cell isolation [51]. Considered to be the most promising stem cells for articular cartilage repair, hUC-MSCs show a higher ability of proliferation and cloning than BM-MSCs and AD-MSCs [52].

## MSCs-based treatment of OP

**Fig. 1:**
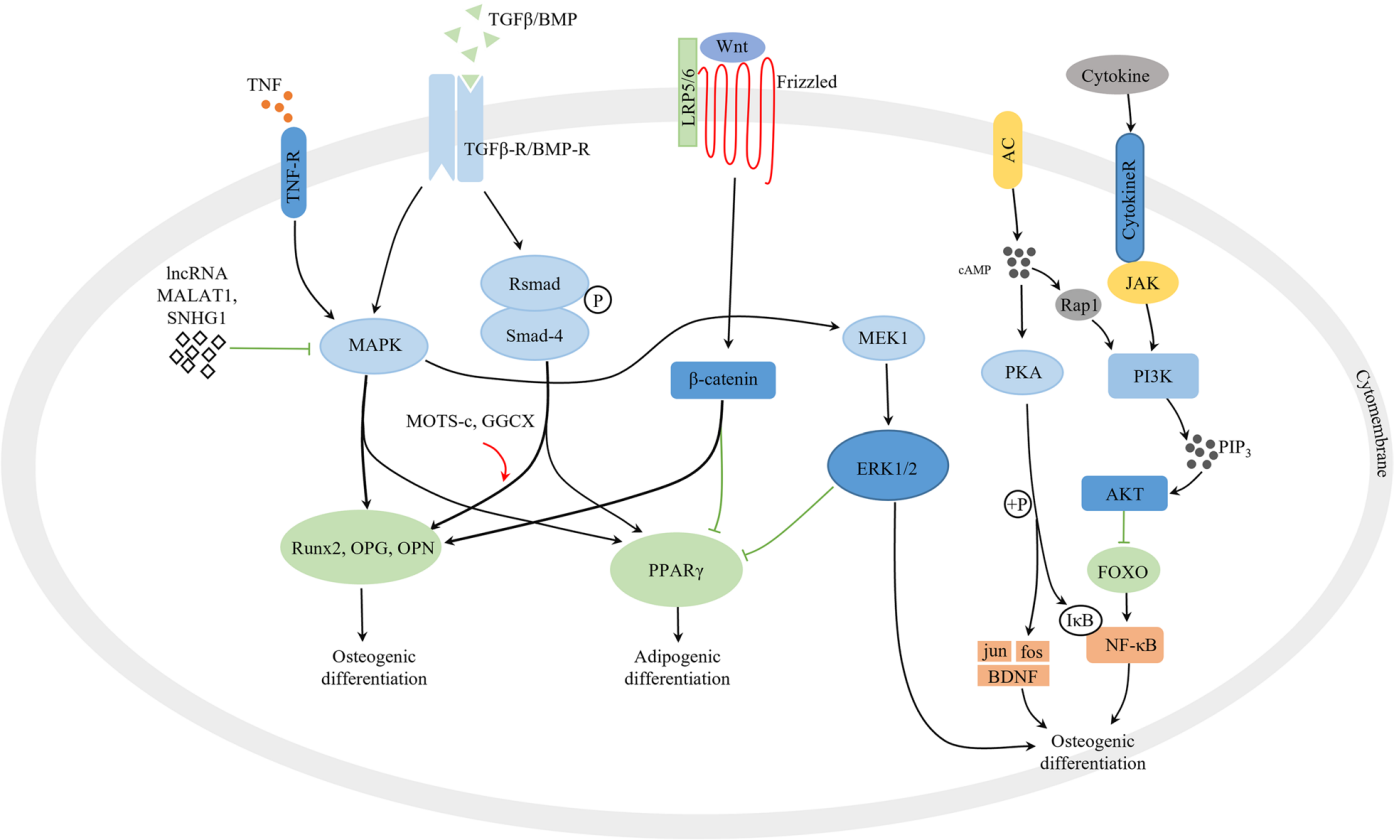
Schematic diagram illustrating mechanisms of osteogenic and adipogenic differentiation of MSC

The incidence of the fractures caused indirectly by OP still accounts for the highest in the elderly population, even though remarkable progresses have been made in the development of OP medications. A present, certain drug and nondrug therapies are mainly performed to treat OP by improving bone strength and preventing fracture [53]. Although they are administered on the patient with OP, these medications have some limitations and even cause adverse reactions. As in the case of MSC transplantation which has been proven to be a feasible and novel therapeutic approach in regenerative medicine and stem cell therapy. Stem cells can differentiate into target tissues by self-differentiation, and promote tissue progenitor cells to differentiate into target tissues by secreting various proteins, enzymes and factors. Through the direct interaction between cells to promote the differentiation of other cells into target tissues, these factors enable stem cells to repair damaged tissues [54]. A growing number of researchers have begun to explore the potential of MSCs in the treatment of chronic diseases [55]. In the current review, therefore, we illustrated the mechanisms of osteogenic and adipogenic differentiation of MSC (Fig. 1) and evaluated potential of bone marrow, umbilical cord, and adipose-derived mesenchymal stem cells in the treatment of OP (Table 1).

**Table 1:** Summary of previous studies on osteogenic differentiation of MSCs

Author, reference	Cell type	Animal model	Condition	Intervention	Consequence
Takada et al. [60]	BMSCs	SAMP6 mice	Irradiated	Injection	BM microenvironment normalized and trabecular bone increased
Ocarino Nde M.et al. [61]	BMSCs	Female Wistar rats	Ovariectomy	Injection	Trabecular bone percentage increased
Huang et al. [62]	BMSCs	Sprague–Dawley rats	Femoral fracture	Injection	Larger callus size and higher mechanical property
Hu et al. [64]	BMSCs	Rats	Osteogenic differentiation	Treated with MOTS-c	MOTS-c promoting osteogenic differentiation via TGF-β/Smad pathway
Wang et al. [65]	BMSCs	Sprague–Dawley rats	Ovariectomy	Transfected with pcDNA-GGCX	Reduced GGCX inhibiting osteogenic differentiation via TGFβ/smad pathway
Ren et al. [66]	BMSCs	Rats	Ovariectomy	Treated with PAAE	PAAE inducing osteogenic differentiation via BMP-2/Smad1,5/Runx2 pathway
Feng et al. [67]	BMSCs	Sprague–Dawley rats	Ovariectomy	Treated with simvastatin	Simvastatin promoting osteogenic differentiation via BMP-2/Smads pathway
Zhao et al. [68]	BMSCs	Rats	MSC-Exo	Co-culture of MSC-Exo and hFOB 1.19	MSC-Exo promoting osteogenic differentiation via MAPK pathway
Chen et al. [79]	AD-MSCs	Patients	Subculture	Osteogenic induction	Osteogenic differentiation of AD-MSCs less affected by age and multiple passage
Wang et al. [81]	AD-MSCs	Female C57BL/6 mice	Ovariectomy	Osteogenic induction	Osteogenic potential of AD-MSCs impaired in osteoporotic mice
Ye et al. [82]	AD-MSCs	Rabbits	Ovariectomy	Osteogenic induction	Stimulating osteogenic differentiation and enhancing bone regeneration
Jin et al. [84]	hAD-MSCs	Mice	Ovariectomy	Knockdown or overexpression of HSPB7 by lentivirus transfection	HSPB7 negatively regulating osteogenic differentiation of hAD-MSCs
Zhou et al. [86]	AD-MSCs	AD-MSCs	Oxidative stress	Transinfected with Let-7c inhibitor	Let-7c inhibiting osteogenic differentiation of AD by targeting SCD-1
Ding et al. [88]	AD-MSCs	Canines	Bone defect	Treated with AD-MSCs plus PRF	PRF enhancing osteogenic potential of AD-MSCs
Na et al. [99]	hUC-MSCs	hUC-MSCs	hUC-MSCs	Treated with GSI-I	GSI-I reducing osteogenic differentiation of hUC-MSCs
Qu et al. [100]	hUC-MSCs	SD rats	Fracture nonunion	AKT blocker injection	AKT modulating osteogenesis induced by hUC-MSCs
Liu et al. [101]	UC-MSCs	Mice	CIA	Transplantation	Upregulating the impaired osteogenic differentiation ability in CIA mice
Liang et al. [104]	hUC-MSCs	Aged rats	Age-related osteoporosis	Treated with secretome	Secretome from hUC-MSCs having the capacity to recover stem cell potential
Wang et al. [108]	hUC-MSCs	hBMSCs	Without OIM	Treated with secretion factors	Initiating osteogenic differentiation of hBMSCs without OIM
Hendrijantini et al. [111]	hUC-MSCs	Female Wistar rats	Ovariectomy	Injection	Increasing osteogenic differentiation and osteoporotic mandibular bone density

BMSCs, bone marrow mesenchymal stem cells; SAMP6, senescence-accelerated mouse prone 6; BM, bone marrow; MOTS-c, mitochondrial open reading frame of the 12S rRNA-c; GGCX, γ-glutamyl carboxylase; PAAE, pilose antler aqueous extract; Runx2, runt-related transcription factor 2; Exo, exosome; MAPK, mitogen-activated protein kinase; AD-MSCs, adipose-derived mesenchymal stem cells; HSPB7, heat shock protein B7; SCD-1, stearoyl-CoA desaturase 1; MFX, microfracture; PRF, platelet-rich fibrin; GSI-I, γ-secreatase inhibitor I; CIA, collagen-induced arthritis; OIM, osteogenic induction medium

### Bone marrow-derived mesenchymal stem cells

BM-MSCs have various biological advantages in terms of tissue repair, and their high osteogenic differentiation, the means of which are widely applied to the repair of bone and cartilage injuries [56]. Previous studies have mainly focused on clarifying the positive role of BM-MSCs in promoting osteogenesis [57, 58]. The efficacy of BM-MSCs in the treatment of OP has been demonstrated in quite a few preclinical animal models, as indicated in the previously reported two studies where both the haemopoietic system and the bone marrow microenvironment were normalized after the direct infusion of allogeneic bone marrow cells into the bone marrow cavity of irradiated SAMP6 mice, and the levels of IL-6, RANKL and IL-11, involved in regulating bone reconstruction, returned to normal, similar to those of B6 mice, thereby ameliorating the imbalance between bone formation and resorption [59, 60]. Moreover, the intraosseous injection of allograft BM-MSCs into the femur in a postmenopausal rat model of OP definitely caused the femoral trabecula of the treated rats to increase significantly within two months, appearing similar to the femurs of the controls. GFP labelling results also confirmed the presence of transplanted BM-MSCs on the trabecular surface of the treated rats [61]. In the Sprague–Dawley adult rat model, of closed transverse fracture with internal fixation, a systemic MSC injection and local MSC injection were performed at the fracture site on the 4th day after fracture; the results of weekly X-ray, micro-CT, mechanical tests, etc., showed that the callus size grew significantly larger in the rats after treatment, but no difference in fracture healing was found between the two regimens administered [62].

With the research furthering on BM-MSC-mediated bone tissue repair, bone marrow aspirate concentrate (BMAC), which is rich in cytokines, has been widely used in the treatment of knee osteoarthritis and other diseases [63]. According to the review of the relevant studies, researchers have come to believe that the body can regulate the function of BM-MSCs through multiple signalling pathways, including the TGF β/Smad pathway [64, 65], BMP-2/Smad pathway [66, 67], MAPK pathway [68, 69], Wnt/β-catenin pathway [70], PPAR-γ pathway [71], etc., and that the body can either promote their osteogenic differentiation or inhibit adipogenic differentiation to regulate OP. As indicated in a previously reported study where the expression of Foxf1 was detected in various tissues of OVX mice and its expression increased significantly in bone extracts and BMSCs, followed by a gradual decrease during the osteogenic differentiation of BM-MSCs, the results of in vitro loss-of-function approach showed that Foxf1 could regulate the osteoblast differentiation of BM-MSCs through the Wnt/β-catenin signalling pathway [72].

Recent researches indicate that each type of factors mainly mediates the cell signal transduction of the corresponding signalling pathways to regulate the function of BM-MSCs. The in vivo and in vitro studies have shown that p38α deficiency can affect the synthesis of RANKL, M-CSF, or other molecules involved in the regulation of osteoclast formation, and that p38α expressed by BM-MSCs can effectively regulate osteogenesis through the TAK1-NF-κB signalling pathway and promote the production of OPG by stem cells to regulate the generation of osteoclasts [73]. Furthermore, the influence of RNA in regulating the osteogenic differentiation of BM-MSCs, in which microRNAs [74] and lncRNAs [75] were aberrantly expressed and combined with their respective target proteins in the patients with OP, thus playing a role in the regulation of osteogenic differentiation of BMSCs.

### Adipose tissue-derived mesenchymal stem cells

Known as pluripotent stem cells, adipose-derived mesenchymal stem cells are one of the common types of MSCs in cell therapy and regenerative medicine. AD-MSCs, mainly isolated from adipose tissues, are more abundant in brown adipose tissues than in white adipose ones. Mostly AD-MSCs which emerge from the mesoderm can be directed to differentiate into other mesoderm-derived tissues such as fat, bone, muscle, tendon, and blood vessel [76]. When the inflammation or injury occurs in vivo, the body may reduce the activity of AD-MSCs, affecting their immune function by regulating paracrine function and differentiation potential [77]. However, the inflammatory cytokines and chemokines released at the injury site can enable AD-MSCs to be recruited there, showing strong local functions in immunoregulation, tissue repair or other processes [51].

Similar to BM-MSCs, AD-MSCs have a high amplification capacity and multilineage differentiation potential [78]; moreover, they are present in adipose tissue, thus producing the advantages of being relatively easy and safe to obtain and low immunogenicity, and their proliferation and differentiation potential are less likely to be affected by the age of the donor and the number of passages [79, 80]. Therefore, the human adult adipose tissue may be the most suitable source of MSCs. As confirmed in the previously reported studies on ovariectomized mouse models [81], rabbit models [82], and in vitro osteogenic experiments involving AD-MSCs [79, 83], AD-MSC-based cell transplant therapy can effectively promote the recovery of OP in the short term and enhance bone regeneration in vivo. Further studies have revealed that osteogenic inducible factors in humans can regulate human adipose-derived stem cells through the ERK pathway [84], PKA pathway [85], and Wnt/β-catenin pathway [86], which significantly improves the osteogenic differentiation potential of AD-MSCs.

All this indicates that AD-MSCs have the promising potential to become a major source for cell transplantation therapy of OP in the future. Nevertheless, the attempts clinically made to inject AD-MSCs into where OP is present in the patient, are prohibitively difficult and of little clinical utility. Therefore, few research teams have been registered to conduct clinical trials of AD-MSCs, locally or systemically, to treat OP patients.

In recent years, however, it has been gratifying that AD-MSCs have been reported to show good results in the clinical treatment of joint microfractures [87], bone defects [88], osteoarthritis [89]. In the prospective randomized trial, autologous AD-MSCs were injected into the joints of patients with knee osteoarthritis for treatment, the therapeutic results showing a significant improvement in the patients’ joint mobility, pain, and bone and cartilage regeneration within 12 months, and indicating the adverse events to be comparable between the experimental group and control in the subsequent follow-ups [89]. From the preclinical study on AD-MSCs and their clinical applications to some diseases, a clear demonstration has been shown on their safety and reliability in vivo [90].

In view of which, we also believe that AD-MSC-based cell transplant therapy can be performed as a novel therapeutic approach to various orthopaedic diseases, including fractures and cartilage destruction. As for the application to OP and to other systemic bone diseases, however, quite a number of preclinical studies are still needed to explore the dosage, mode of administration and adverse reactions of the drug in the clinical applications.

### Human umbilical cord-derived mesenchymal stem cells

Human umbilical cord mesenchymal stem cells are mainly derived from umbilical cord blood, Wharton’s jelly, blood vessels and their surrounding tissues [91]. An early study showed that many pluripotent MSCs could be isolated from UC samples, of which the cells obtained from umbilical cord blood were the most abundant, and UC-MSCs were more abundant in preterm cord blood than in term blood [92]. Since then, UC-MSCs have become another important source for the isolation of mesenchymal stem cells thanks to their nonharmful acquisition mode and few related ethical issues [93].

Biologically, UC-MSCs show advantages over bone marrow and adipose-derived stem cells, in terms of low immunogenicity, high proliferation and differentiation, and excellent anti-inflammatory and other immunomodulatory functions [94–96]. Since 2009, when researchers began to commit themselves to exploring the therapeutic potential of the allogeneic transplantation of UC-MSCs, the successful potential of UC-MSCs in the animal models and of their clinical application has been promoted [97]. UC-MSCs, as a potential cell source for cartilage and bone regeneration, are mainly regulated by Wnt [98], Notch [99], and AKT signalling [100], among other signalling pathways, as well as by cytokines such as TNF-α [101] that promote the differentiation of MSCs towards the osteogenic lineage.

Recent studies have suggested that the efficacy of UC-SMCs in disease treatment mainly depends on cell secretion [102], and the concentration of active factors secreted by UC-MSCs is 10–100 times that of factors secreted by BM-MSCs under the same culture conditions [103]. From these cells the secreted active molecules facilitate the restoring of viable cell proliferation and differentiation, and contribute to bone remodelling in the bone metabolism system. It has been confirmed that the secretory group of hUC-MSCs, producing TGF-β, FGF, VEGF, EGF, etc., has the ability to restore stem cell potential and delay local bone loss in age-related OP [104]. Nowadays, the application of UC-MSCs to orthopaedic diseases is mainly related to the repair of articular cartilage, as indicated in a clinical randomized controlled trial which demonstrated that the patients who had received a local injection of allogeneic UCMSCs showed an improvement in pain and function, without serious adverse events [105]. Also, UC-MSCs have achieved a good curative effect in treating rheumatoid arthritis and a good therapeutic effect in repairing bone tissue defects, as indicated in a recent retrospective study on the clinical efficacy of hUC‑MSCs in osteonecrosis of the femoral head (ONFH), which suggested that arterially infused hUC‑MSCs could migrate via the blood to the area of femoral head necrosis and differentiate into osteoblasts; 24 months later, these cells could significantly reduce the necrotic volume of the femoral head [106]. Moreover, in a study on the application of bone particles combined with hUC-MSCs to repair rabbit lumbar bone defects, hUC-MSCs, when transplanted into the defect area of the vertebral lamina and foot arch, promoted effectively bone regeneration there [107]. It was suggested that the loss of stem cell function was closely related to the occurrence of OP, and that UC-MSCs could present a higher therapeutic potential than MSCs derived from other tissues [108].

Additionally, the secretion patterns of MSCs from different tissues may be different, the differences indicating specificity for different diseases [109]. In the perimenopausal rat models, hUC-MSC therapy could significantly delay ovarian aging, thus improving the ovarian reserve function [110], and the osteogenic effect of hUC-MSC therapy could significantly increase the bone density of the osteoporotic mandible [111]. In the case of AD-MSCs, we still believe that UC-MSCs have a promising potential of treating OP clinically, although they have not yet been registered for the clinical trials of OP.

## Current challenges and safety issues

Currently, MSCs are generally considered safe and effective in the animal models, and in the clinical trials, no major adverse events have been reported. Admittedly, the stem cells have the potential of self-renewal and multilineage differentiation as a unique biological characteristic; however, they can give rise to such safety problems as the potential for vascular injury [112], vascular embolism [113], infection, immune responses and abnormal accumulation of amyloid-β [114], of which the most worrisome is the development of tumours induced by chromosomal abnormalities. In a rat stroke model, cerebral blood flow decreased while the embolic events and associated lesion size increased in the rats after the intra-arterial cell infusion of MSCs; the occurrence of these phenomena seemed to be related to the cell dose and the velocity of cell infusion [115]. In a randomized multicentre clinical trial where 53 patients with refractory rheumatoid arthritis were treated with allogeneic AD-MSCs, a total of 141 side effects were observed, including 1 severe lacunar infarction [116]. Due to the characteristics of MSCs and the interference of external conditions, the occurrence of tumours cannot be completely avoided. In an early study in which mouse cancer models were used, the immune cells including neutrophils, macrophages and mast cells, were found to express MMP-9, contributing to the occurrence of skin squamous cell carcinogenesis [117]. Furthermore, MSCs were reported to be regulated by a variety of cytokines and chemokines in tumour tissues, mediating cell proliferation, angiogenesis, and metastasis [118]. Evidence suggests that MSCs can inhibit the growth of tumour cells in a healthy microenvironment and that only after acquiring tumour-like gene mutations can secrete factors to promote tumour progression [119]. In the lung adenocarcinoma (A549 cells) experiments conducted in vitro, soluble factors secreted by MSCs in the tumour microenvironment could promote the growth and metastasis of cancer cells [120]. The reduced tumorigenesis is hypothesised to be related to the methylation of genes in MSCs, as indicated in the research where DNA methylation in mesenchymal stem cells was found to be capable of regulating the expression and activation of PTEN, promoting osteogenic effects and reducing tumorigenesis [121]. Similarly, the abnormal DNA methylation in MSCs could affect the progression of myeloma, and the treatment of abnormal DNA methylation could exert an antimyeloma and osteogenic effect [122].

The clinical application of MSCs has another problem, at present, no consensus has been reached on the best single dose of MSC injection in clinical practice. In the treatment of osteoarthritis, focal cartilage defects, etc., studies have suggested that a single high dose (1.0 × 10^8^) administered on the patients with osteoarthritis can significantly improve the clinical symptoms and therapeutic outcomes of radiology and arthroscopy [123]. Surely, the efficiency of MSC transplantation needs to be examined in the clinical context. In the study of bone tissue repair, intramedullary injected MSCs could reach the bone marrow quickly, but the apoptosis rate was significantly high during the migration to the damaged site; therefore, the ultimate number of MSCs at the damaged site was significantly small [124]. Although ideal results have been achieved in the animal models in which OP was treated, the systemic involvement of OP and the existence of adverse events associated with MSCs still restrict its clinical application to OP treatment.

## Conclusion

OP is a systemic metabolic bone disease characterized by low BMD caused by abnormal bone metabolism. At present, the clinical approaches mainly involve exercise intervention and drug therapy, the former delaying bone loss in the early stage of the disease, and the latter having a therapeutic effect; however, long-term drug use can cause serious side effects. So far, great progresses have been achieved in the treatment of clinical diseases with stem cells, which are characterized by unique biological advantages in terms of proliferation, differentiation, and immune regulation, among other properties. However, there are still many challenges to be addressed in the practical application of MSC therapy. Overall, MSC-based stem cell therapy is relatively safe for the treatment of local bone injury. Actually, the potential of this method in OP treatment is unlimited, but the fate, tumorigenicity and ethical problems of clinical treatment of stem cells limit its clinical application. Further preclinical research is needed to clarify its long-term safety and effectiveness. With a growing understanding of exosomes, we speculate that stem cell-derived exosomes could be used as a feasible strategy to replace stem cell therapy to fight osteoporosis. Therefore, we suggest that in the near future researches much importance should be attached to the clinical transformation of mesenchymal stem cell therapy and the stem cell-derived exosomes in the treatment of OP.

## Data Availability

Not applicable.

## Abbreviations

OP: Osteoporosis
BM: Bone marrow
BMD: Bone mineral density
MSCs: Mesenchymal stem cells
BM-MSCs: Bone marrow-derived mesenchymal stem cells
AD-MSCs: Adipose tissue-derived MSCs
UC-MSCs: Umbilical cord-derived MSCs
BMI: Body mass index
BMAC: Bone marrow aspirate concentrate
RANKL: Receptor activator of nuclear factor-κ B ligand
OPG: Osteoprotegerin
OVX: Ovariectomized
PTH: Parathyroid hormone
LRP5/6: LDL receptor related protein 5/6
M-CSF: Macrophage-colony stimulating factor
RNA: Ribonucleic acid
DNA: Deoxyriboncleic acid
lncRNAs: Long non-coding RNAs
TGF-β: Transforming growth factor-β
FGF: Fibroblast growth factor
EGF: Epidermal growth factor
VEGF: Vascular epidermal growth factor
ONFH: Osteonecrosis of the femoral head
